# Expression of HLA Class I and HLA Class II by Tumor Cells in Chinese Classical Hodgkin Lymphoma Patients

**DOI:** 10.1371/journal.pone.0010865

**Published:** 2010-05-28

**Authors:** Xin Huang, Anke van den Berg, Zifen Gao, Lydia Visser, Ilja Nolte, Hans Vos, Bouke Hepkema, Wierd Kooistra, Sibrand Poppema, Arjan Diepstra

**Affiliations:** 1 Department of Pathology and Medical Biology, University Medical Center Groningen, University of Groningen, Groningen, The Netherlands; 2 Department of Pathology, Health Science Center, Peking University, Beijing, China; 3 Unit of Genetic Epidemiology and Bioinformatics, Department of Epidemiology, University Medical Center Groningen, University of Groningen, Groningen, The Netherlands; 4 Department of Laboratory Medicine, University Medical Center Groningen, University of Groningen, Groningen, The Netherlands; Leiden University Medical Center, Netherlands

## Abstract

**Background:**

In Caucasian populations, the tumor cells of Epstein Barr virus (EBV)-positive classical Hodgkin Lymphomas (cHL) patients more frequently express HLA class I and HLA class II molecules compared to EBV-negative cHL patients. HLA expression (in relation to EBV) in Asian cHL patients has not been previously investigated.

**Methodology/Principal Findings:**

We randomly selected 145 cHL patients with formalin-fixed, paraffin embedded tissue blocks available from 5 hospitals from the Northern part of China. Hematoxylin & Eosin-stained slides were used to reclassify the histological subtypes according to the WHO classification. EBV status was determined by visualization of EBERs in tumor cells using in situ hybridization. Membranous expression of HLA molecules was detected by immunohistochemistry using antibodies HC-10 (class I heavy chain) and anti-ß2-microglobulin for HLA class I, and CR3/43 for HLA class II. EBV+ tumor cells were observed in 40% (58/145) of the cHL patients. As expected, the percentage of EBV+ cases was much higher in the mixed cellularity subtype (71%) than in the nodular sclerosis subtype (16%) (p<0.001). Expression of HLA class I was observed in 79% of the EBV+ cHL cases and in 30% of the EBV- cases (p<0.001). For HLA class II, 52% of EBV+ cHL cases were positive, compared to 43% in EBV- cases (p = 0.28).

**Conclusions:**

The results in the Northern China population were similar to those in the Caucasian population for HLA class I, but not for HLA class II.

## Introduction

Classical Hodgkin lymphoma (cHL) is a malignant neoplasm of the immune system, characterized by a minority of B cell derived tumor cells, named Hodgkin Reed-Sternberg cells (HRS cells) and numerous reactive cells consisting of lymphocytes, histiocytes, eosinophils, and plasma cells. The HRS cells are large, sometimes bi- or multinucleated cells with prominent nucleoli and a characteristic CD20 negative to weakly positive, CD30+ and CD15+/− immunophenotype [1]. However, the presence of HRS cells in an abundant inflammatory infiltrate indicates that anti-tumor immune responses apparently are insufficient for the eradication of HRS cells. It has been shown that the tumor cells of cHL employ several mechanisms to escape from immune responses, even more so in Epstein Barr virus (EBV) associated cases [2]–[4]. EBV has been acknowledged as the major infectious agent causing cHL, although the proportion of EBV associated cHL varies from 20% to nearly 100% in different populations [3], [5]. In addition, the proportion of EBV+ cases is also age-dependent with a first high incidence peak in children and a second peak in adults around age 60 [3], [5]. EBV-infected HRS cells consistently express a limited set of proteins, consisting of latent membrane protein 1 (LMP1), latent membrane protein 2 (LMP2) and EBV nuclear antigen 1 (EBNA1) [5]. Antigenic peptides derived from these three proteins can be processed and presented by the human leukocyte antigen (HLA) class I and class II pathways, the efficiency of which largely depends on the peptide binding affinity of the highly polymorphic HLA alleles [6], [7] [8], [9]. Cytotoxic T lymphocytes (CTLs) are known to be the primary effector cells to eradicate EBV-infected B cells that present LMP1 and LMP2 antigenic peptides in the context of appropriate HLA class I molecules [6], [7]. In addition, there's *in vitro* evidence that EBV infection and the related malignant transformation are controlled by CD4+ T cells, depending on HLA class II restricted antigen presentation [10]. In other words, both HLA class I-restricted CTL responses and HLA class II-restricted CD4+ T-cell responses are essential for a successful anti-tumor immune defense. Therefore, downregulation of HLA class I and HLA class II antigens might be implicated in the pathogenesis of cHL by allowing tumor cells to escape host immunosurveillance.

Several research groups have studied the association between HLA expression and cHL in the Western population [11]–[16], but nothing is known for the Asian population. Since HLA types are known to widely differ between Caucasians and Asians, we set out to investigate the expression of HLA molecules in Chinese cHL cases for drawing comparison between the two populations. We studied HLA class I as well as HLA class II expression in relation to EBV status in a population from the Northern part of China.

## Materials and Methods

### Patient material

Formalin-fixed paraffin-embedded tissue blocks of lymph node biopsies from 145 cHL patients were obtained from 5 hospitals in northern China (Dept. of Pathology, Health Science Center, Peking University; Dept. of Pathology, First Hospital of Jilin University; Dept. of Pathology, Shougang Hospital, Peking University; Dept. of Pathology, Beijing Air Army General Hospital; Zhanye Regional Hospital, Gansu Province). The biopsies were stained with hematoxylin & eosin (H&E) and histopathological subtyping was performed according to the WHO classification.

### *In situ* hybridization

Detection of EBV in tumor cells was performed by *in situ* hybridization (ISH) on paraffin sections with a fluorescein-conjugated PNA probe specific for the EBV-encoded EBER RNAs (DAKO, Glostrup, Denmark). A known EBV+ tissue section was used as a positive control.

### Immunohistochemical staining

4-µm thick paraffin sections were deparaffinized by xylene and rehydrated through a graded ethanol series into water. Microwave antigen retrieval was performed with Tris-EDTA solution (10mM Tris Base, 1mM EDTA Solution, PH 9.0) and endogenous peroxidase activity was blocked in 3% H_2_O_2_. The expression of HLA class I was detected using monoclonal antibody HC-10 at a dilution of 1∶200 (kindly provided by Prof. dr. J. Neefjes, the Netherlands Cancer Institute, Amsterdam), which recognizes HLA B and C molecules, as well as a few HLA-A molecules [17]. In addition, the polyclonal rabbit anti human ß2-microglobulin (DAKO) at a dilution of 1∶200 was used as an additional marker to detect HLA class I. For detection of HLA class II, we used the CR3/43 monoclonal antibody (DAKO) that binds to a specific monomorphic epitope in the ß chain of HLA-DP, HLA-DQ and HLA-DR. All antibodies were detected using a standard Avidin Biotin Complex (ABC) immunoperoxidase method. Diaminobenzidine was used as the chromogen and hematoxylin was used for counterstaining.

### Evaluation of HLA class I and class II staining

HLA class I heavy chain (HC-10) staining was scored simultaneously with ß2-microglobulin staining. The same scoring rules were used for HLA class II. The surrounding inflammatory cells were used as an internal positive control and also as a reference for assessing the intensity of HLA expression by HRS cells. A strong membranous staining on at least 50% of the tumor cells was identified as positive. In case the staining intensity on the tumor cells was similar to the intensity on the surrounding reactive cells, membranes in between adjacent tumor cells were evaluated.

### Statistical analysis

HLA expression was determined in relation to EBV status. Differences between EBV+ and EBV-neg groups in relation to HLA expression as well as several clinicopathologic variables were assessed by Chi square test, Fisher's exact test or Mann Whitney U test. The correlation between HLA class I and class II expression was evaluated with Chi square test. In addition, multivariate analysis using logistic regression was performed to adjust for confounders. The data were analyzed with SPSS for windows, version 16.0. A p-value <0.05 was considered significant.

## Results

### Clinicopathologic features

145 patients diagnosed with cHL were subdivided into histological subtypes according to the WHO classification. Subtype could not be unequivocally determined in 18% (n = 26), usually because there was not enough tissue to properly evaluate the background architecture. These patients were classified as cHL, not otherwise specified (NOS). In the remaining patients the nodular sclerosis (NS) subtype was the most common one, accounting for 63% (n = 75) of patients, followed by mixed cellularity (MC) with 35% (n = 42) of patients. The lymphocyte rich (LR) subtype was rare (n = 2) and the lymphocyte depleted subtype was absent. The median age of the patients at the time of diagnosis was 28 years, ranging from 4 to 74 years. There was a clear male predominance with a male to female ratio of 2∶1.

### EBV status and clinicopathologic variables

**Figure 1:**
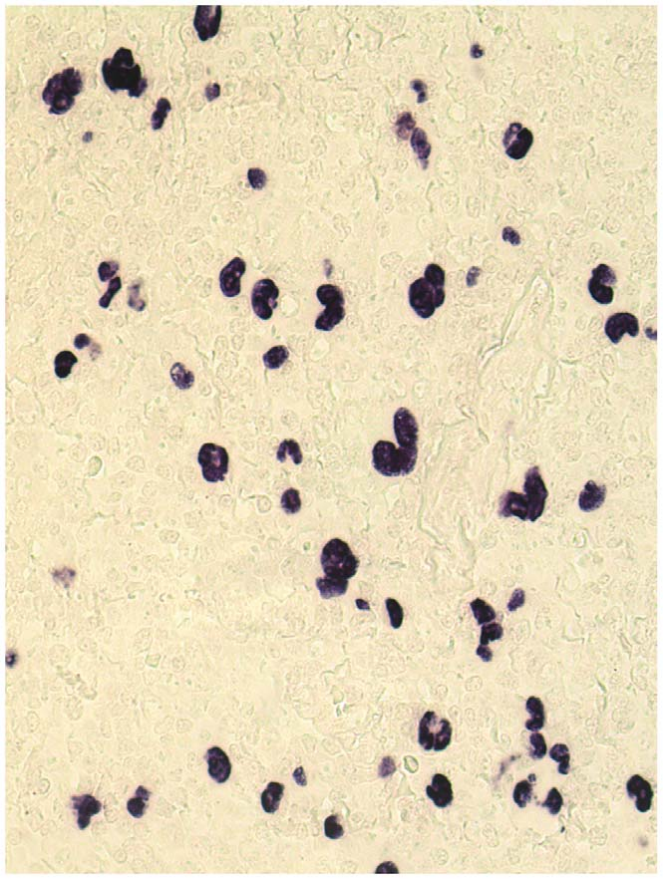
*In situ* hybridization (ISH) for EBERs in an EBV+ cHL case. The EBER-ISH revealed a homogeneous positive signal in the nucleus of all Hodgkin and Reed–Sternberg (HRS) cells as well as in some small reactive cells (original magnification ×40).

EBERs in HRS cells were demonstrated in 40% of the patients (n = 58). In these patients, all tumor cells showed consistent nuclear labeling (see figure 1). A low number of positive small bystander cells were observed in some cases.

Subtype and sex demonstrated statistically significant differences between EBV+ and EBV-neg cases. As expected, the MC subtype showed the highest percentage of EBV+ cases (30 of 42 cases [71%]). In addition, males more frequently had EBV+ cHL than females (48% compared to 24%). In terms of patients' age, no significant difference was found between EBV-associated and non- EBV-associated cHL (Table 1).

**Table 1:** Distribution of age, sex and histology by EBV status.

	All patientsn = 145	EBV-Positiven = 58	EBV-Negativen = 87	P
**median age** (range)	28(4–74)	31 (4–74)	28 (8–74)	0.56‡
**Sex**				
Male	66.2% (n = 96)	79.3% (n = 46)	57.5% (n = 50)	0.006*
Female	33.7% (n = 49)	20.7% (n = 12)	42.5% (n = 37)	
**Histological subtype**				
NS	51.7% (n = 75)	20.7% (n = 12)	72.4% (n = 63)	<0.001†
MC	29.0% (n = 42)	51.7% (n = 30)	13.8% (n = 12)	
LR	1.4% (n = 2)	3.4% (n = 2)	0% (n = 0)	
NOS	17.9% (n = 26)	24.2% (n = 14)	13.8% (n = 12)	

‡ Mann Whitney U test.

* Chi square test.

† Fisher's exact test.

NS indicates nodular sclerosis; MC, mixed cellularity; LR, lymphocyte rich; NOS, not otherwise specified.

### HLA class I and HLA class II expression

**Figure 2:**
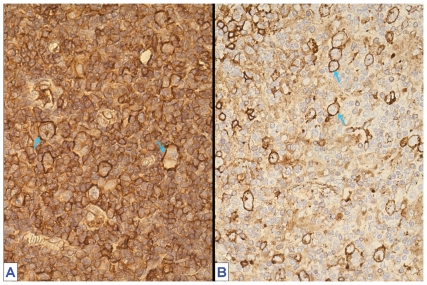
Immunohistochemical detection of Human Leukocyte Antigen (HLA) expression on formalin-fixed paraffin-embedded tissue sections of classical Hodgkin lymphoma (cHL). (A) HLA class I (using the HC-10 antibody) expression in cHL. All cells show immunoreactivity on the membrane. Hodgkin and Reed–Sternberg (HRS) cells stand out with their stronger signal (arrow) (original magnification 40×). (B) HLA class II (CR3/43) expression in cHL. Most HRS cells are positive (arrow), surrounded by negative inflammatory cells (original magnification ×40).

Expression of HLA class I heavy chains was consistent with that of ß2-microglobulin and the rate of positivity was 50% (n = 72). In most patients with HLA class I positive tumor cells, the HRS cells showed a higher staining intensity than the reactive background cells, especially in EBV+ cases (see figure 2A). For HLA class II expression by tumor cells, 46% of patients (n = 67) were positive. Usually, the HRS cells were surrounded by HLA class II negative reactive cells (see figure 2B).

In 26% of patients (n = 38) there was co-expression of HLA class I and class II, whereas in 30% of patients (n = 44) the tumor cells were double negative. Although there was a trend for HLA class I negative cases to also be HLA class II negative, and vice versa, this correlation was not statistically significant (Table 2).

**Table 2:** Correlation between HLA class I and class II expression.

	HLA class I		P‡
	Positive n = 72	Negative n = 73	
**HLA class II**			
Positive (n = 67)	52.3% (n = 38)	39.7% (n = 29)	0.115
Negative (n = 78)	47.7% (n = 34)	60.3% (n = 44)	

‡ Chi square test.

### Correlation between expression of HLA, EBV status and clinicopathologic variables

The results of HLA class I and HLA class II expression in relation to EBV status, the different histological subtypes, sex and age are summarized in Table 3. Expression of HLA class I was significantly more frequent in EBV+ than in EBV-neg cases (P<0.001). Histological subtypes correlated with HLA class I expression, with the highest frequency of positive expression in the MC subtype (32/42 = 76%) and the lowest in the NS subtype (22/75 = 29.3%) (P<0.001). Using multivariate logistic regression analysis a significant effect of EBV status on HLA class I expression was observed after adjusting for histological subtype (P<0.001). However subtype did not remain significant when correcting for EBV status implying that EBV status explained the observed association between histological subtype and HLA class I expression. In contrast, HLA class II expression was not associated with EBV or subtype. Neither HLA class I nor HLA class II expression was found to correlate with patients' age. In addition, our data showed that Chinese female cHL patients more frequently maintained expression of HLA class II (P = 0.01), but not expression of HLA class I.

**Table 3:** HLA class I and class II expression by HRS cells in relation to EBV status, histology, sex and median age.

	HLA class I		P‡	HLA class II		P‡
	Positiven = 72	Negativen = 73		Positiven = 67	Negativen = 78	
**EBV**						
Pos.	64% (n = 46)	16% (n = 12)	<0.001	45% (n = 30)	36% (n = 28)	0.277
Neg.	36% (n = 26)	84% (n = 61)		55% (n = 37)	64% (n = 50)	
**Histology**						
NS	31% (n = 22)	72% (n = 53)	<0.001*	51% (n = 34)	53% (n = 41)	0.955*
MC	44% (n = 32)	14% (n = 10)		27% (n = 18)	31% (n = 24)	
LR	3% (n = 2)	0% (n = 0)		1% (n = 1)	1% (n = 1)	
NOS	22% (n = 16)	14% (n = 10)		21% (n = 14)	15% (n = 12)	
**Sex**						
Male	67% (n = 48)	66% (n = 48)	0.907	55% (n = 37)	75% (n = 59)	0.01
Female	33% (n = 24)	34% (n = 25)		45% (n = 30)	25% (n = 19)	
**median age** (range)	33 (4∼74)	26 (6∼61)	0.246†	28 (4∼74)	28 (4∼74)	0.666†

‡ Chi square test.

* Fisher's exact test, NOS histology not included.

† Mann Whitney U test.

NS indicates nodular sclerosis; MC, mixed cellularity; LR, lymphocyte rich; NOS, not otherwise specified.

### Comparison between Chinese and Dutch cHL patients

The data from the current Chinese population were compared with data from a population based study in the Netherlands, performed by our group [12]. EBV positivity and MC subtype were more common in Chinese cHL patients than in Dutch cHL patients (40% vs. 33% and 29% vs. 11%, respectively). In the Chinese cHL patients, the proportion of children was higher (age<18: 22.8% vs. 9.2%) while that of the elderly was lower (age>60: 9.0% vs. 15.8%). A similar association of HLA class I expression with EBV status was observed in both populations. However, expression of HLA class II highly correlated with HLA class I in the Dutch population, which was not the case in Chinese patients. Loss of HLA class II expression by HRS cells was more common in Chinese compared to Dutch patients (54% vs. 41%). This difference was independent of sex, EBV status and histological subtype, shown using a multivariate logistic regression model (P = 0.012). Table 4 shows that in Dutch patients the expression of HLA class II was strongly associated with EBV positivity (P = 0.005). Also, the NS subtype was more frequently deficient in HLA class II expression than the MC subtype (P = 0.016). Neither of these associations was observed in the Chinese cHL patients. However, only in the Chinese cHL patients an association between sex and HLA class II expression was observed (P = 0.01).

**Table 4:** Comparison of the association of HLA class II expression with EBV status and histological subtypes between Dutch* and Chinese cHL patients.

	Dutch patients*		P‡	Chinese patients		P‡
	HLA class II			HLA class II		
	Positive (n = 171)	Negative (n = 121)		Positive (n = 67)	Negative (n = 78)	
**EBV**						
Positive	39.8% (n = 68)	24.0% (n = 29)	0.005	44.8% (n = 30)	35.9% (n = 28)	0.277
negative	61.2% (n = 103)	76.0% (n = 92)		55.2% (n = 37)	64.1% (n = 50)	
**Histology** $						
NS	84.1% (n = 143)	87.3% (n = 103)	0.016†	64.1% (n = 34)	62.1% (n = 41)	0.796†
MC	14.7% (n = 25)	5.1% (n = 6)		34.0% (n = 18)	36.4% (n = 24)	
LR	0.6% (n = 1)	3.4% (n = 4)		1.9% (n = 1)	1.5% (n = 1)	
LD	0.6% (n = 1)	4.2% (n = 5)		0	0	
**Sex**						
Male	57.9% (n = 99)	58.7% (n = 71)	0.89	55.2% (n = 37)	75.6% (n = 59)	0.01
Female	42.1% (n = 72)	41.3% (n = 50)		44.8% (n = 30)	24.4% (n = 19)	
**Median age** (range)	34 (8∼88)	32 (8∼88)	0.85	28 (4∼74)	28 (4∼74)	0.666#

‡ Chi square test.

* Data have been published (Diepstra A et al (12)).

† Comparison was made between NS and MC subtype only.

# Mann Whitney U test.

$ Classical Hodgkin lymphoma not otherwise specified excluded.

NS indicates nodular sclerosis; MC, mixed cellularity; LR, lymphocyte rich; LD, lymphocyte depleted.

## Discussion

This study involved the largest group of northern Chinese cHL patients evaluated for expression of HLA class I and HLA class II by HRS cells. HLA class I expression was strongly associated with EBV-positivity, similar to the Caucasian populations. However, an association between HLA class II expression and EBV status was not apparent, in contrast to the data from Western Europe.

The EBV association with cHL has been investigated by several research groups within different geographic locales and ethnicities, showing a common association between EBV-associated cHL, male sex and MC subtype [3], [5]. These associations were also present in this northern Chinese patient population.

Although the pathogenesis of cHL is still not clearly established, immune escape mechanisms seem to play an important role, especially in cases that are EBV-associated. Downregulation of HLA on the surface of tumor cells has been observed in various types of human malignancies, including cHL [18]. Our present data demonstrate that deficiency in membranous expression of HLA antigen by tumor cells is quite common in EBV-neg cHL, since 49.7% and 46.2% cases expressed HLA class I or class II respectively and only 26.2% cases expressed both. Lack of HLA expression is generally thought to be advantageous for the persistence of tumor cells by escaping T cell mediated immunosurveillance.

An intriguing finding is that HLA class I expression is usually retained in EBV+ cases, although HLA class I-restricted CTL responses in general play a major role in eliminating virus infection. This correlation is consistent with the results of four previous studies performed on Caucasian cHL populations from the Netherlands and the United Kingdom [13], [14], [16]. Our data also show a correlation between HLA class I expression and MC subtype, which can be attributed to the close relationship of EBV involvement with MC subtype. It is apparent that not only the quantity, but also the quality of HLA molecules determines the ability to mount an immune response. The composition of HLA alleles determines the peptide-binding ability and the subsequent immune response. A recent report on a detailed investigation within the HLA region lends proof to this concept, as patients with EBV-associated cHL more frequently carry HLA-A*01 in comparison to EBV-negative cHL patients who usually carry HLA-A*02 instead [11], [15]. It should be noted that persistence of HLA class I expression can be advantageous for the tumor cells to survive Natural Killer cell-mediated lysis [19].

HLA class II-restricted CD4 T cell immune responses are largely, but not exclusively restricted to exogenous antigens and it has become evident that these responses are essential for an efficient eradication of EBV infection [10]. Our group previously reported a significant association of HLA class II expression with EBV status in 292 Dutch cHL patients. In contrast, we did not find this association in the Chinese population in the current study, although we could see a slight non-significant tendency to maintain the expression of HLA class II in EBV-associated cHL.

In addition, the previously reported correlation between the expression of HLA class I and class II [12] was absent in the present study. A major difference between the Dutch and Chinese population is the percentage of MC subtype patients with 10.6% and 28.9% of patients respectively. Strikingly, the expression of HLA class II in MC subtype patients was very different between the two populations and was much more frequently downregulated in the Chinese patients. Since we used the same antibody, staining protocols and scoring criteria for evaluating HLA class II expression in both populations, the differential expression likely reflects a biological difference between the two different populations which presumably relates to HLA-based genetic heterogeneity. The HLA system is extremely polymorphic and allelic frequencies vary dramatically between racial groups. A certain HLA allele that is relatively uncommon in one population can be highly prevalent and associated with a specific disease in another. In the Chinese population one or more prevalent HLA class II allele(s) might present immunodominant EBV antigenic peptides to the immune system, thereby exerting selection pressure to downregulate this molecule. Thus, yet-to-be-defined ethnic-specific HLA alleles are likely to affect the strength of association between HLA class II expression and the strongly EBV associated MC subtype. It should be noted that Oudejans et al [16] also studied the expression of HLA class II in Dutch cHL patients, but did not find the same association. However, they used another antibody, recognizing HLA-DR only, the sample size of their study was much smaller (n = 63) and they used a different set of scoring criteria [16].

An additional association found in the Chinese population was a strong correlation between downregulation of HLA class II with male sex. Since male sex is associated with EBV+ tumor cell status in general, this might partially explain the lack of association of HLA class II with EBV status in the Chinese patients. However, there is no obvious explanation for the association of HLA class II expression with sex. Interestingly, lack of HLA class II expression is related to inferior failure free survival in the Dutch population [12]. An important consequence of the differences in HLA class II expression between the Chinese and Dutch patients might be that this adverse prognostic impact of HLA class II absence is not necessarily present in other populations.

In conclusion, our data demonstrate that in northern Chinese patients, EBV+ cHL tumor cells more frequently retain HLA class I expression, similar to Caucasian populations. However, the association of HLA class II expression with positive EBV status, as observed in Caucasians, is not present in the northern Chinese population. Investigation at the molecular level is needed to further explore the role of anti-tumor immune responses in the pathogenesis of cHL. Differences in ethnic background should be taken into account and might explain discrepancies in incidence pattern, EBV association and other aspects in different populations.
